# Acute kidney injury and 30-day unplanned cirrhosis readmissions: A nationwide Readmissions Database retrospective cohort study

**DOI:** 10.1097/MD.0000000000051220

**Published:** 2026-10-23

**Authors:** Asad Ali Ahmed Cheema, Eliza Aisha, Muhammad Munhib Shehzad, Ayesha Naveed, Ayesha Wasim, Syed Muhammad Ibrahim Shah Bokhari, Yaseen Ali Khan, Fatima Jawed, Muhammad Usman Hashmi, Ahsan Raza Raja, Sajeela Khan, Haider Imran

**Affiliations:** a Department of Medicine, International School of Medicine, International University of Kyrgyzstan, Bishkek, Kyrgyzstan; b Department of Medicine, Dow University of Health Sciences, Karachi, Pakistan; c Department of Medicine, University College of Medicine and Dentistry, University of Lahore, Lahore, Pakistan; d Department of Medicine, Shalamar Medical and Dental College, Lahore, Pakistan; e Department of Medicine, King Edward Medical University, Mayo Hospital, Lahore, Pakistan; f Department of Medicine, Fazaia Medical College, Islamabad, Pakistan; g Department of Medicine, Shahida Islam Medical Complex, Shahida Islam Teaching Hospital, Lodhran, Pakistan; h Department of Medicine, Letterkenny University Hospital, Letterkenny, Donegal, Ireland; i Department of Medicine, Aga Khan University, Karachi, Pakistan; j Department of Family Medicine, MCPS Family Medicine Trained, Aga Khan University, Karachi, Pakistan; k Department of Medicine, Foundation University Medical College, Islamabad, Pakistan.

**Keywords:** acute kidney injury, liver cirrhosis, Nationwide Readmissions Database, unplanned readmission

## Abstract

30-day unplanned readmissions after hospitalization for liver cirrhosis are common. Acute kidney injury (AKI) may be associated with higher early readmission risk. Using the Nationwide Readmissions Database 2016 to 2017, we identified adults with an index cirrhosis admission from January through November by International Classification of Diseases Tenth Revision Clinical Modification (ICD-10-CM) codes. AKI was defined by N17.x during the index stay. Unplanned 30-day readmissions were identified after excluding planned readmissions and trauma-related hospitalizations. Survey-weighted multivariable logistic regression adjusted for patient, clinical, and hospital factors. Among 1,68,816 index admissions, AKI occurred in 25.6%. Overall, 25.0% had unplanned 30-day readmission, higher with AKI than without (26.0% vs 24.2%; absolute difference 1.8 percentage points). AKI was independently associated with readmission (adjusted odds ratio 1.10, 95% confidence interval 1.05–1.16). AKI admissions had longer length of stay (median 6 vs 4 days), higher inflation-adjusted charges ($58,507 vs $32,587), and nonhome discharge (34% vs 16%; all *P* < .001). Readmissions occurred at a median of 12 days and had 7.72% in hospital mortality. Ascites and chronic kidney disease were strong predictors. AKI was associated with readmission among patients without chronic kidney disease but not among those with it (interaction *P* < .001). AKI during the index admission was associated with unplanned 30-day readmission, although the association was modest in magnitude, and marked a high-risk cirrhosis population with greater resource use. Because of the observational, retrospective design, causality cannot be established.

## Graphical abstract

**Figure:**
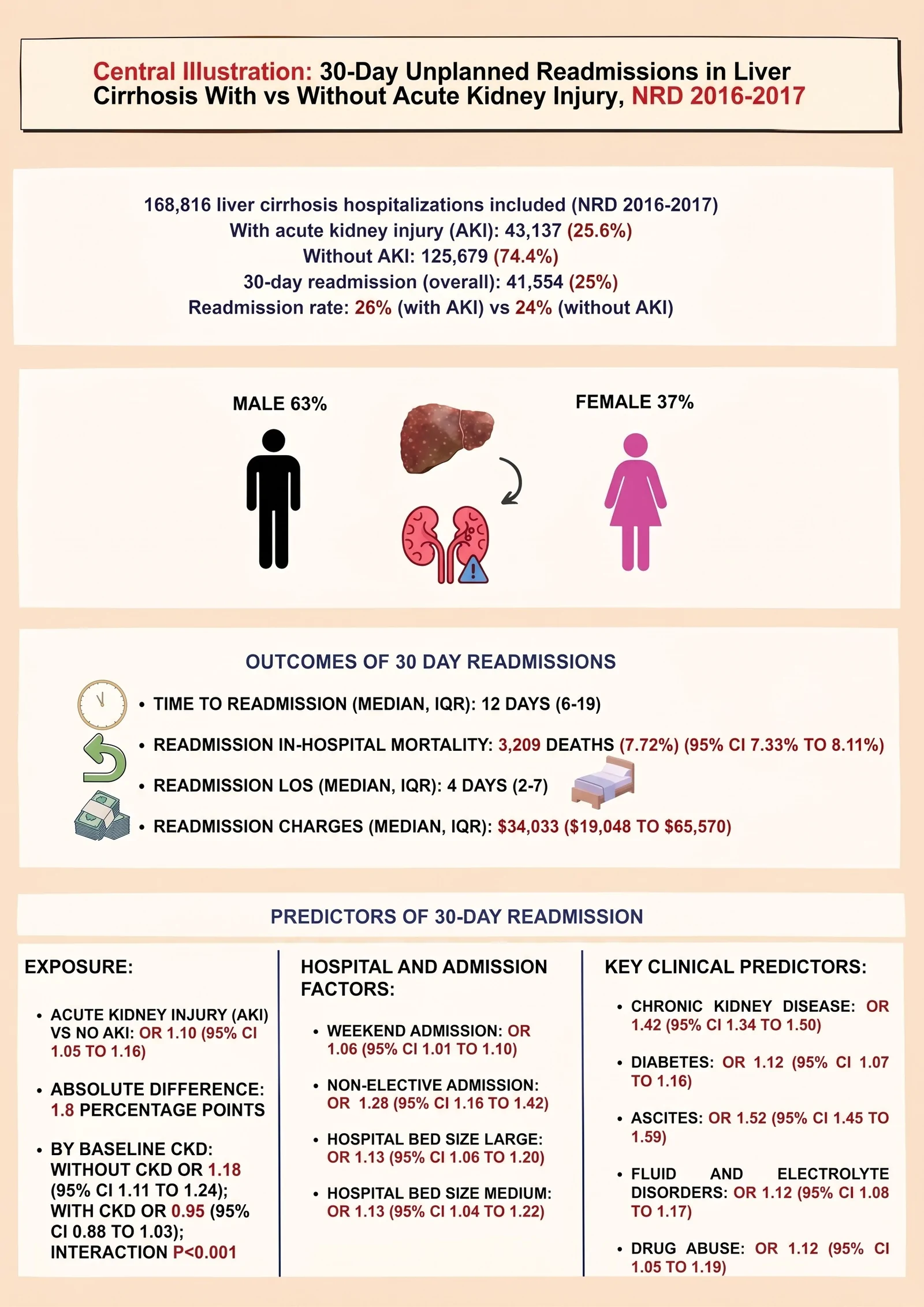

**Key Points**

- Acute kidney injury affected 25.6% of 1,68,816 index liver cirrhosis hospitalizations in the Nationwide Readmissions Database 2016 to 2017.
- Unplanned 30-day readmission occurred in 25.0% overall and was higher with acute kidney injury than without (26.0% vs 24.2%).
- Acute kidney injury remained independently associated with unplanned 30-day readmission after survey-weighted multivariable adjustment (adjusted odds ratio 1.10, 95% confidence interval 1.05–1.16), corresponding to an absolute difference of 1.8 percentage points.
- The association was present among patients without preexisting chronic kidney disease but not among those with it (interaction *P* < .001).
- Acute kidney injury was associated with higher resource utilization and adverse readmission outcomes, including longer index length of stay, higher charges, more nonhome discharge, early readmission (median 12 days), and 7.72% in-hospital mortality during readmission.

## 1. Introduction

Hospital readmissions within 30 days of discharge are a central United States quality metric and a major driver of spending.^[1,2]^ In Medicare fee for service, 19.6% of beneficiaries discharged from hospital in 2003 and 2004 were rehospitalized within 30 days, and unplanned rehospitalizations were estimated to cost Medicare $17.4 billion in 2004.^[1]^ Under the Centers for Medicare and Medicaid Services Hospital Readmissions Reduction Program, payments are reduced for hospitals with excess risk standardized unplanned 30-day readmissions, with the payment reduction capped at 3%.^[2]^ In 2016, chronic liver disease related hospitalizations accounted for $18.8 billion in total inpatient costs, and the chronic liver disease hospitalization rate was 3757 per 1,00,000 hospitalizations.^[3]^ Among patients hospitalized with cirrhosis, reported 30-day readmission rates range from 12.9% in a multistate all payer cohort to 31.4% in a Nationwide Readmissions Database (NRD) analysis, and a systematic review estimated a pooled 30-day readmission rate of 26% with a 95% confidence interval (CI) from 22% to 30%.^[4-6]^

Cirrhosis is characterized by recurrent decompensation events that frequently precipitate hospitalization and early return to hospital.^[7]^ Ascites is commonly the first decompensation defining event, with 5% to 10% of patients with compensated cirrhosis developing ascites per year.^[7]^ The development of ascites is associated with a reduction in 5-year survival from 80% to 30%.^[7]^ Acute kidney injury (AKI) is a common and high impact complication in this setting, with an incidence ranging from 20% to 50% among cirrhosis hospitalizations for acute decompensation.^[8]^ Consensus guidance emphasizes that AKI in cirrhosis is associated with markedly increased morbidity and mortality and requires standardized recognition and evaluation.^[9]^ Socioeconomic factors further shape outcomes in cirrhosis. In addition, patients residing in the lowest income quartile have 32% higher odds of hospital readmission than those in the highest income quartile.^[10]^

Prior national analyses of cirrhosis readmission have generally evaluated broad predictor sets rather than a designated exposure.^[4-6,10]^ A prior NRD analysis using the same principal-diagnosis cirrhosis cohort did not report AKI among its independent predictors of 30-day readmission.^[6]^ Where AKI has been quantified it has appeared as 1 covariate within a wider model and in cohorts defined by a specific decompensating complication, with an approximately 20% increase in readmission risk in decompensated cirrhosis,^[11]^ an adjusted odds ratio (OR) of 1.11 (95% CI: 1.00–1.22) in cirrhosis complicated by hepatic encephalopathy,^[12]^ and a hazard ratio of 1.14 in variceal bleeding.^[13]^ AKI has not been evaluated as a prespecified exposure in an ICD-10-era cohort defined by a principal diagnosis of cirrhosis with planned readmissions excluded. Separate evaluation is warranted because AKI is frequently reversible, its precipitants are often modifiable, and it is among the few index-admission findings that are directly actionable at discharge. Using the 2016 and 2017 NRD, we examined whether AKI during the index hospitalization for cirrhosis is independently associated with unplanned 30-day readmission and quantified related in hospital mortality, length of stay (LOS), and total hospitalization charges (TOTCHG).^[14,15]^

## 2. Methods

### 2.1. Reporting guideline

This observational cohort study is reported in accordance with the Strengthening the Reporting of Observational Studies in Epidemiology (STROBE) statement.^[16]^

### 2.2. Study design and data source

We conducted a retrospective cohort study using the 2016 and 2017 NRD, developed as part of healthcare cost and utilization project (HCUP) by the Agency for Healthcare Research and Quality. The NRD is an all-payer, encounter-level administrative database constructed from HCUP State Inpatient Databases and contains verified patient linkage numbers that can be used to track individuals across hospitals within a State during a calendar year.^[15]^ Beginning in 2016, diagnoses and procedures in the NRD are coded using the International Classification of Diseases, Tenth Revision, Clinical Modification and Procedure Coding System (ICD-10-CM/PCS).^[14]^ Because the NRD is a calendar-year database, patients cannot be followed across different calendar years.^[15]^

### 2.3. Study population and case identification

Adult patients aged 18 years or older were eligible if they had an index hospitalization for liver cirrhosis during calendar years 2016 to 2017. The index hospitalization was defined as the first eligible inpatient admission per patient, per calendar year, with a principal diagnosis of liver cirrhosis, identified using ICD-10-CM codes K74.3, K74.4, K74.5, K74.6, and K70.3. For patients with multiple cirrhosis-related admissions, subsequent hospitalizations were excluded from being treated as index events and were instead evaluated as potential readmissions.

AKI during the index hospitalization was ascertained using ICD-10-CM code N17.x. Index hospitalizations were categorized based on the presence or absence of AKI.

To ensure complete 30-day follow-up within the same calendar year, we excluded patients with an index discharge date in December. Consequently, only hospitalizations with a discharge date between January 1 and November 30 of each calendar year were eligible for inclusion as index events.^[17]^

### 2.4. Outcomes

The primary outcome was 30-day unplanned all-cause readmission following discharge from the index hospitalization. Readmissions were identified using NRD patient linkage variables and HCUP readmission methodology.^[17]^ Planned readmissions were excluded using the HCUP Planned Readmission Algorithm to focus on unplanned readmissions.^[18]^ Trauma-related hospitalizations were excluded to minimize misclassification of injury-related admissions.

Secondary outcomes during the index hospitalization included length of stay (LOS), TOTCHG, and discharge disposition (home vs nonhome). Among patients who experienced a 30-day readmission, readmission-level outcomes included LOS, TOTCHG, and time to readmission. Charges were inflation adjusted to 2017 US dollars using the Consumer Price Index.^[19]^

HCUP reporting requirements prohibit publication of tabulated cell sizes less than or equal to 10 and allow reporting only counts of 11 or more. Therefore, in-hospital mortality counts were not reported when doing so would result in a cell size <11.^[20]^

### 2.5. Covariates

Patient- and hospital-level covariates were extracted from the NRD. Demographic and socioeconomic variables included age, sex, patient residence, primary expected payer (Medicare, Medicaid, private insurance, or other), and ZIP code-based median household income quartile. Hospital characteristics included weekend admission status, hospital bed size (small, medium, or large), and hospital location and teaching status (urban teaching, urban nonteaching, or rural).

Clinical comorbidities were identified using the HCUP Elixhauser Comorbidity Software for ICD-10-CM,^[21]^ which includes hypertension, diabetes mellitus, CKD, congestive heart failure, anemia, coagulopathy, chronic pulmonary disease (CPD), alcohol abuse, drug abuse, depression, obesity, and fluid and electrolyte disorders. Additionally, cirrhosis-related complications assessed during the index hospitalization included ascites, esophageal varices, portal hypertension, hepatorenal syndrome (HRS), and sepsis. Index-stay factors such as LOS and discharge disposition were also included.

### 2.6. Statistical analysis

All analyses accounted for the NRD complex survey design using discharge weights and design variables to generate nationally representative estimates.^[15]^ Baseline characteristics and index admission outcomes were summarized by AKI status. *P* values were obtained from design-based statistical tests using the Rao–Scott adjusted chi-square test for categorical variables and design-based Kruskal–Wallis tests for continuous variables. Absolute survey-weighted standardized mean differences (SMD) were calculated for each baseline characteristic in Table 1 (for categorical variables with more than 2 levels, a multivariate SMD across levels), with SMD > 0.10 considered a meaningful imbalance. Missingness was low (<1% for all analytic variables except median household income quartile, 1.7%). Multivariable models used complete cases, and no multiple imputation was performed. Baseline tables report percentages among non-missing values.

**Table 1:** Baseline characteristics.

Characteristic	Overall N = 1,68,816*	Without acute kidney injury N = 1,25,679*	With acute kidney injury N = 43,137*	*P*-value†	SMD‡
Age (yr)	57 (50, 64)	56 (49, 64)	59 (52, 66)	<.001	0.209
Sex				.031	0.018
Male	1,06,384 (63%)	78,916 (63%)	27,468 (64%)		
Female	62,432 (37%)	46,763 (37%)	15,669 (36%)		
Median income quartile				<.001	0.067
0–25th percentile	58,086 (35%)	43,910 (36%)	14,175 (33%)		
26th to 50th percentile	45,910 (28%)	34,359 (28%)	11,552 (27%)		
51st to 75th percentile	37,432 (23%)	27,514 (22%)	9918 (23%)		
76th to 100th percentile	24,641 (15%)	17,719 (14%)	6922 (16%)		
Weekend admission				.002	0.026
Monday–Friday	1,30,555 (77%)	96,852 (77%)	33,703 (78%)		
Saturday–Sunday	38,262 (23%)	28,828 (23%)	9434 (22%)		
Type of admission				.017	0.037
Elective	8311 (4.9%)	6440 (5.1%)	1871 (4.3%)		
Nonelective	1,60,200 (95%)	1,19,033 (95%)	41,167 (96%)		
Hospital bed size				<.001	0.108
Small	22,113 (13%)	17,196 (14%)	4916 (11%)		
Large	1,02,532 (61%)	74,686 (59%)	27,846 (65%)		
Medium	44,172 (26%)	33,798 (27%)	10,374 (24%)		
Hospital teaching status				<.001	0.179
Metropolitan, nonteaching	40,601 (24%)	32,035 (25%)	8567 (20%)		
Metropolitan, teaching	1,19,119 (71%)	86,186 (69%)	32,933 (76%)		
Nonmetropolitan	9095 (5.4%)	7458 (5.9%)	1637 (3.8%)		
Insurance				<.001	0.179
Medicaid	51,445 (31%)	39,857 (32%)	11,588 (27%)		
Medicare	61,826 (37%)	44,027 (35%)	17,799 (41%)		
Other	18,323 (11%)	14,750 (12%)	3573 (8.3%)		
Private	36,826 (22%)	26,782 (21%)	10,043 (23%)		
Hypertension	75,075 (44%)	54,219 (43%)	20,857 (48%)	<.001	0.105
Diabetes	39,154 (23%)	28,964 (23%)	10,190 (24%)	.2	0.014
Chronic kidney disease	30,305 (18%)	13,728 (11%)	16,578 (38%)	<.001	0.673
Coagulopathy	79,518 (47%)	55,996 (45%)	23,522 (55%)	<.001	0.200
Anemia	1,07,111 (63%)	76,291 (61%)	30,820 (71%)	<.001	0.228
Chronic pulmonary disease	30,419 (18%)	22,697 (18%)	7722 (18%)	.7	0.004
Congestive heart failure	20,047 (12%)	12,984 (10%)	7063 (16%)	<.001	0.178
Alcohol abuse	1,16,064 (69%)	86,985 (69%)	29,079 (67%)	<.001	0.039
Drug abuse	13,650 (8.1%)	10,842 (8.6%)	2808 (6.5%)	<.001	0.080
Depression	23,672 (14%)	17,706 (14%)	5966 (14%)	.5	0.007
Obesity	19,661 (12%)	13,873 (11%)	5788 (13%)	<.001	0.073
Fluid and electrolyte disorders	91,062 (54%)	58,840 (47%)	32,222 (75%)	<.001	0.596
Ascites	1,27,994 (76%)	91,911 (73%)	36,082 (84%)	<.001	0.258
Esophageal varices	64,661 (38%)	48,505 (39%)	16,156 (37%)	.015	0.024
Portal hypertension	78,617 (47%)	57,226 (46%)	21,391 (50%)	<.001	0.081
Hepatorenal syndrome	11,755 (7.0%)	1979 (1.6%)	9776 (23%)	<.001	0.683
Sepsis	5590 (3.3%)	1756 (1.4%)	3834 (8.9%)	<.001	0.344

* Median (Q1, Q3); n (%).

† Design-based Kruskal–Wallis test; Pearson’s χ²: Rao & Scott adjustment.

‡ SMD = absolute survey-weighted standardized mean difference (with vs. without acute kidney injury); SMD > 0.10 suggests meaningful imbalance.

Survey-weighted multivariable logistic regression was used to evaluate independent predictors of 30-day unplanned readmission, including demographic, clinical, hospital-level, and index-stay factors. Covariates were selected a priori rather than through data-driven selection, based on established associations with cirrhosis decompensation and 30-day readmission in prior literature, biologic plausibility, and availability of validated ICD-10-CM-based ascertainment, to limit overfitting in the survey-weighted model and preserve interpretability.^[5,6,10,22]^ Results are reported as ORs with 95% CIs. Among readmitted patients, time to readmission and readmission-level LOS and charges were summarized using survey-weighted methods. Analyses were performed in R version 4.5.0 (R Foundation for Statistical Computing, Vienna, Austria) using the survey and srvyr packages.

## 3. Results

### 3.1. Study cohort and 30-day unplanned readmissions

Using the 2016 and 2017 NRD, we identified 1,68,816 weighted adult index hospitalizations with a principal diagnosis of liver cirrhosis. AKI was present in 43,137 (25.6%) index hospitalizations and absent in 1,25,679 (74.4%; Table 1). Overall, 41,554 (25.0%) index hospitalizations were followed by an unplanned 30-day readmission. Readmission occurred more frequently after index hospitalizations with AKI than after those without AKI (26% vs 24%, *P* < .001; Table 2).

**Table 2:** Outcomes of index hospitalization.

Characteristic	Overall N = 1,68,816*	Without Acute Kidney Injury N = 1,25,679*	With Acute Kidney Injury N = 43,137*	*P*-value†
30-Day readmission	41,554 (25%)	30,351 (24%)	11,203 (26%)	<.001
Length of stay (d)	4.0 (2.0, 7.0)	4.0 (2.0, 6.0)	6.0 (4.0, 12.0)	<.001
Inflation-adjusted total charges ($)	36,976 (21,200, 68,374)	32,587 (19,314, 56,247)	58,507 (30,886, 1,24,647)	<.001
Discharged to nonhome setting	34,832 (21%)	20,108 (16%)	14,723 (34%)	<.001

* n (%); Median (Q1, Q3).

† Design-based Kruskal–Wallis test; Pearson’s χ²: Rao & Scott adjustment.

### 3.2. Baseline demographic, socioeconomic, and hospital characteristics

The median age of the cohort was 57 years (Q1 50, Q3 64), and 63% were male (Table 1). Patients with AKI were older than those without AKI (median 59 vs 56 years, *P* < .001, SMD 0.21). Among demographic, socioeconomic, and admission characteristics, age, insurance status, and hospital teaching status showed the largest between-group imbalances (standardized mean difference [SMD] 0.21, 0.18 and 0.18, respectively). Medicare coverage was more common among AKI hospitalizations (41% vs 35%), and Medicaid coverage less common (27% vs 32%). AKI hospitalizations occurred more often in metropolitan teaching hospitals (76% vs 69%) and less often in nonmetropolitan hospitals (3.8% vs 5.9%). Hospital bed size was marginally imbalanced (SMD 0.11), with AKI hospitalizations more frequent in large hospitals (65% vs 59%). Differences in sex, weekend admission, type of admission, and median household income quartile reached statistical significance but were negligible in magnitude (all SMD ≤ 0.07; Table 1).

### 3.3. Clinical comorbidity burden and cirrhosis-related complications

Clinical complexity was substantially greater among hospitalizations with AKI (Table 1). The largest between-group imbalances were in HRS (23% vs 1.6%; SMD 0.68), chronic kidney disease (CKD; 38% vs 11%; SMD 0.67), fluid and electrolyte disorders (75% vs 47%; SMD 0.60), and sepsis (8.9% vs 1.4%; SMD 0.34). Moderate imbalances were present for ascites (84% vs 73%; SMD 0.26), anemia (71% vs 61%; SMD 0.23), coagulopathy (55% vs 45%; SMD 0.20), and congestive heart failure (16% vs 10%; SMD 0.18), with a smaller imbalance for hypertension (48% vs 43%; SMD 0.11). Portal hypertension, obesity, drug abuse, alcohol abuse, and esophageal varices differed statistically but not meaningfully (all SMD < 0.10), and diabetes, CPD, and depression differed neither statistically nor in magnitude (Table 1).

### 3.4. Index hospitalization outcomes

Index hospitalization outcomes differed substantially by AKI status (Table 2). LOS was longer for hospitalizations with AKI than without AKI (median 6 vs 4 days, *P* < .001). Inflation-adjusted TOTCHG were higher in the AKI group ($58,507 vs $32,587, *P* < .001). Discharge to a nonhome setting occurred more frequently among AKI hospitalizations (34% vs 16%, *P* < .001; Table 2).

### 3.5. Timing and outcomes of readmission hospitalizations

Among index hospitalizations followed by readmission within 30 days, the median time to readmission was 12 days (Q1 6, Q3 19). Readmission hospitalizations were associated with substantial adverse outcomes and resource use. There were 3209 in-hospital deaths during readmission, corresponding to a readmission in-hospital mortality rate of 7.72% (95% CI: 7.33–8.11%). Median LOS during readmission was 4 days (Q1 2, Q3 7), and median inflation-adjusted total charges during readmission were $34,033 (Q1 $19,048, Q3 $65,570; Table 3).

**Table 3:** Timing and characteristics of 30-day readmission hospitalizations.

Characteristic	Value
Time from index discharge to 30-day readmission, median (IQR), d	12 (6–19)
In-hospital mortality during readmission, n	3209
In-hospital mortality during readmission, %	7.72
In-hospital mortality during readmission, % (95% CI)	7.33–8.11
Readmission length of stay, median (IQR), d	4 (2–7)
Readmission total charges, median (IQR), USD	$34,033 ($19,048–$65,570)

CI = confidence interval; IQR = interquartile range; USD = US dollars.

### 3.6. Multivariable predictors of 30-day unplanned readmission

In survey-adjusted multivariable logistic regression (Table 4), AKI during the index hospitalization remained independently associated with higher odds of 30-day unplanned readmission (OR 1.10, 95% CI: 1.05–1.16, *P* < .001). This corresponded to an absolute difference in unplanned 30-day readmission of 1.8 percentage points (26.0% with AKI vs 24.2% without AKI). Several admission and hospital factors were also associated with increased odds of readmission, including weekend admission (OR 1.06, 95% CI: 1.01–1.10, *P* = .008), nonelective admission (OR 1.28, 95% CI: 1.16–1.42, *P* < .001), and treatment at medium or large hospitals (medium OR 1.13, 95% CI: 1.04–1.22, *P* = .002; large OR 1.13, 95% CI: 1.06–1.20, *P* < .001). Nonmetropolitan hospitals were associated with lower odds of readmission (OR 0.75, 95% CI: 0.68–0.82, *P* < .001), while metropolitan teaching status was not associated with readmission (OR 0.99, 95% CI: 0.94–1.03, *P* = .6; Table 4).

**Table 4:** Multivariable predictors for 30-day readmission.

Characteristic	OR	95% CI	*P*-value
Acute kidney injury categories			
Without acute kidney injury	–	–	
With acute kidney injury	1.10	1.05, 1.16	<.001
Age (yr)	0.99	0.99, 0.99	<.001
Sex			
Male	–	–	
Female	1.00	0.96, 1.04	>0.9
Median income quartile			
0–25th percentile	–	–	
26th to 50th percentile	0.97	0.93, 1.02	.2
51st to 75th percentile	0.94	0.89, 0.98	.010
76th to 100th percentile	0.95	0.90, 1.01	.085
Weekend admission			
Monday–Friday	–	–	
Saturday–Sunday	1.06	1.01, 1.10	.008
Type of admission			
Elective	–	–	
Nonelective	1.28	1.16, 1.42	<.001
Hospital bed size			
Small	–	–	
Large	1.13	1.06, 1.20	<.001
Medium	1.13	1.04, 1.22	.002
Hospital teaching status			
Metropolitan, nonteaching	–	–	
Metropolitan, teaching	0.99	0.94, 1.03	.6
Nonmetropolitan	0.75	0.68, 0.82	<.001
Insurance			
Medicaid	–	–	
Medicare	0.95	0.90, 1.01	.078
Other	0.68	0.64, 0.72	<.001
Private	0.75	0.70, 0.79	<.001
Hypertension			
No	–	–	
Yes	1.02	0.98, 1.06	.3
Diabetes			
No	–	–	
Yes	1.12	1.07, 1.16	<.001
Chronic kidney disease			
No	–	–	
Yes	1.42	1.34, 1.50	<.001
Coagulopathy			
No	–	–	
Yes	0.96	0.92, 0.99	.016
Anemia			
No	–	–	
Yes	1.01	0.97, 1.05	.7
Chronic pulmonary disease			
No	–	–	
Yes	1.05	1.00, 1.11	0.042
Congestive heart failure			
No	–	–	
Yes	0.95	0.90, 1.00	.067
Alcohol abuse			
No	–	–	
Yes	0.94	0.90, 0.97	.002
Drug abuse			
No	–	–	
Yes	1.12	1.05, 1.19	<.001
Depression			
No	–	–	
Yes	1.05	0.98, 1.11	.2
Obesity			
No	–	–	
Yes	0.92	0.87, 0.97	.002
Fluid and electrolyte disorders			
No	–	–	
Yes	1.12	1.08, 1.17	<.001
Ascites			
No	–	–	
Yes	1.52	1.45, 1.59	<.001
Esophageal varices			
No	–	–	
Yes	0.84	0.81, 0.88	<.001
Portal hypertension			
No	–	–	
Yes	0.95	0.92, 0.99	.013
Hepatorenal syndrome			
No	–	–	
Yes	0.88	0.81, 0.95	<.001
Sepsis			
No	–	–	
Yes	0.55	0.49, 0.61	<.001
Discharged to nonhome setting			
No	–	–	
Yes	0.76	0.73, 0.80	<.001
Length of stay	1.00	0.99, 1.00	<.001

CI = confidence interval, OR = odds ratio.

The strongest clinical predictors of readmission were ascites (OR 1.52, 95% CI: 1.45–1.59, *P* < .001) and CKD (OR 1.42, 95% CI: 1.34–1.50, *P* < .001). Both associations were larger in magnitude than that observed for AKI (OR 1.10). Other clinical predictors associated with higher odds included diabetes (OR 1.12, 95% CI: 1.07–1.16, *P* < .001), fluid and electrolyte disorders (OR 1.12, 95% CI: 1.08–1.17, *P* < .001), CPD (OR 1.05, 95% CI: 1.00–1.11, *P* = .042), and drug abuse (OR 1.12, 95% CI: 1.05–1.19, *P* < .001; Table 4). Adjusted ORs for AKI and for the clinical comorbidities and cirrhosis-related complications included in the model are shown in Figure 1.

**Figure 1.**
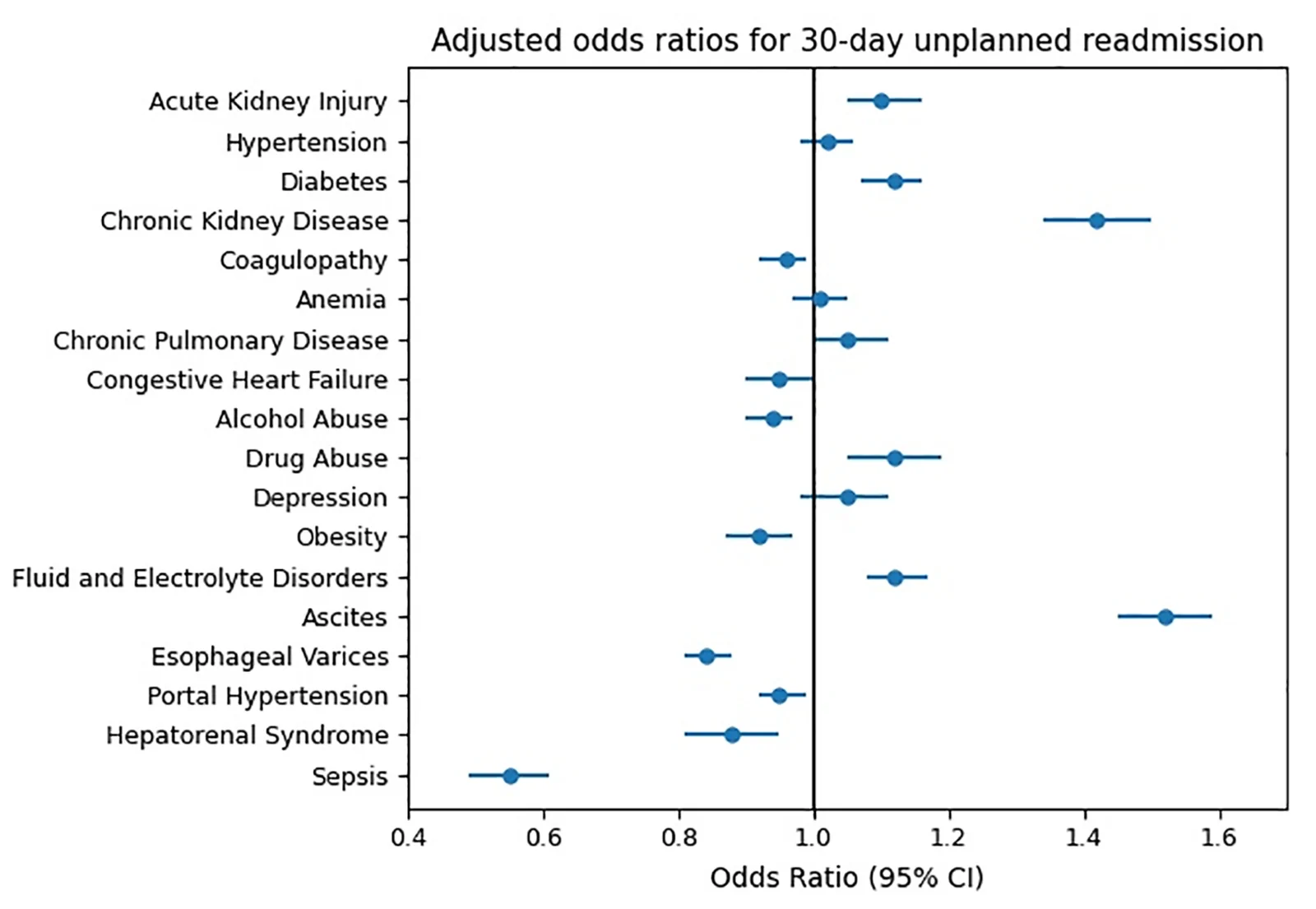
Forest plot showing adjusted odds ratios (95% CI) for acute kidney injury, clinical comorbidities, and cirrhosis-related complications associated with 30-day unplanned readmission. CI = confidence interval.

Several variables were associated with lower odds of readmission in the adjusted model, including sepsis (OR 0.55, 95% CI: 0.49–0.61, *P* < .001), HRS (OR 0.88, 95% CI: 0.81–0.95, *P* < .001), esophageal varices (OR 0.84, 95% CI: 0.81–0.88, *P* < .001), portal hypertension (OR 0.95, 95% CI: 0.92–0.99, *P* = .013), coagulopathy (OR 0.96, 95% CI: 0.92–0.99, *P* = .016), alcohol abuse (OR 0.94, 95% CI: 0.90–0.97, *P* = .002), obesity (OR 0.92, 95% CI: 0.87–0.97, *P* = .002), and discharge to a nonhome setting (OR 0.76, 95% CI: 0.73–0.80, *P* < .001; Table 4). Age was associated with slightly lower odds of readmission per year increase (OR 0.99, 95% CI: 0.99–0.99, *P* < .001; Table 4). LOS had a statistically significant association with readmission, although the magnitude per day was small (OR 1.00, 95% CI: 0.99–1.00, *P* < .001; Table 4).

Several modeled covariates were not statistically significant, including female sex (OR 1.00, 95% CI: 0.96–1.04, *P* > .9), hypertension (OR 1.02, 95% CI: 0.98–1.06, *P* = .3), and Medicare compared with Medicaid (OR 0.95, 95% CI: 0.90–1.01, *P* = .078). By income quartile, the 51st to 75th percentile group had lower odds of readmission compared with the lowest quartile (OR 0.94, 95% CI: 0.89–0.98, *P* = .010), while the 26th to 50th percentile and the highest quartile were not statistically significant (Table 4). Compared with Medicaid, private insurance and other insurance were associated with lower odds of readmission (private OR 0.75, 95% CI: 0.70–0.79, *P* < .001; other OR 0.68, 95% CI: 0.64–0.72, *P* < .001; Table 4).

### 3.7. Sensitivity analyses

After excluding discharge disposition and LOS from the multivariable model, AKI remained significantly associated with 30-day readmission (adjusted OR 1.06, 95% CI: 1.01–1.11), compared with 1.10 (95% CI: 1.05–1.16) in the primary model (Table 5). In models stratified by baseline CKD, AKI was associated with higher odds of readmission among patients without CKD (adjusted OR 1.18, 95% CI: 1.11–1.24) but not among patients with CKD (adjusted OR 0.95, 95% CI: 0.88–1.03); the AKI × CKD interaction was significant (*P* < .001), indicating that baseline CKD modifies the association (Table 6).

**Table 5:** Multivariable predictors for 30-day readmission excluding discharge disposition and length of stay.

Characteristic	OR	95% CI	*P*-value
Acute kidney injury categories			
Without acute kidney injury	–	–	
With acute kidney injury	1.06	1.01, 1.11	.010
Age (yr)	0.99	0.98, 0.99	<.001
Sex			
Male	–	–	
Female	1.00	0.96, 1.04	>.9
Median income quartile			
0–25th percentile	–	–	
26th to 50th percentile	0.97	0.93, 1.02	.2
51st to 75th percentile	0.94	0.89, 0.98	.010
76th to 100th percentile	0.95	0.89, 1.00	.068
Weekend admission			
Monday–Friday	–	–	
Saturday–Sunday	1.05	1.01, 1.10	.014
Type of admission			
Elective	–	–	
Nonelective	1.27	1.15, 1.40	<.001
Hospital bed size			
Small	–	–	
Large	1.13	1.06, 1.20	<.001
Medium	1.13	1.04, 1.22	.003
Hospital teaching status			
Metropolitan, nonteaching	–	–	
Metropolitan, teaching	0.99	0.94, 1.04	.6
Nonmetropolitan	0.74	0.68, 0.82	<.001
Insurance			
Medicaid	–	–	
Medicare	0.95	0.90, 1.00	.038
Other	0.68	0.64, 0.73	<.001
Private	0.75	0.71, 0.80	<.001
Hypertension			
No	–	–	
Yes	1.02	0.98, 1.06	.2
Diabetes			
No	–	–	
Yes	1.12	1.07, 1.17	<.001
Chronic kidney disease			
No	–	–	
Yes	1.42	1.34, 1.50	<.001
Coagulopathy			
No	–	–	
Yes	0.95	0.91, 0.98	.003
Anemia			
No	–	–	
Yes	1.00	0.96, 1.04	>.9
Chronic pulmonary disease			
No	–	–	
Yes	1.05	1.00, 1.10	.075
Congestive heart failure			
No	–	–	
Yes	0.93	0.88, 0.99	.017
Alcohol abuse			
No	–	–	
Yes	0.93	0.89, 0.97	<.001
Drug abuse			
No	–	–	
Yes	1.11	1.04, 1.18	.002
Depression			
No	–	–	
Yes	1.03	0.97, 1.10	.3
Obesity			
No	–	–	
Yes	0.92	0.87, 0.97	.003
Fluid and electrolyte disorders			
No	–	–	
Yes	1.10	1.06, 1.14	<.001
Ascites			
No	–	–	
Yes	1.52	1.45, 1.58	<.001
Esophageal varices			
No	–	–	
Yes	0.85	0.81, 0.88	<.001
Portal hypertension			
No	–	–	
Yes	0.96	0.92, 0.99	.016
Hepatorenal syndrome			
No	–	–	
Yes	0.83	0.77, 0.90	<.001
Sepsis			
No	–	–	
Yes	0.48	0.43, 0.53	<.001

CI = confidence interval, OR = odds ratio.

**Table 6:** Multivariable predictors for 30-day readmission stratified by baseline chronic kidney disease.

Characteristic	Without CKD			With CKD		
	OR	95% CI	*P*-value	OR	95% CI	*P*-value
Acute kidney injury categories						
Without acute kidney injury	–	–		–	–	
With acute kidney injury	1.18	1.11, 1.24	<.001	0.95	0.88, 1.03	.2
Age (yr)	0.99	0.99, 0.99	<.001	0.98	0.98, 0.99	<.001
Sex						
Male	–	–		–	–	
Female	0.97	0.93, 1.02	.2	1.10	0.99, 1.22	.076
Median income quartile						
0–25th percentile	–	–		–	–	
26th to 50th percentile	0.98	0.93, 1.03	.4	0.96	0.87, 1.06	.4
51st to 75th percentile	0.95	0.90, 1.00	.058	0.91	0.81, 1.01	.080
76th to 100th percentile	0.98	0.92, 1.04	.5	0.87	0.77, 0.98	.027
Weekend admission						
Monday-Friday	–	–		–	–	
Saturday-Sunday	1.06	1.01, 1.11	.011	1.05	0.96, 1.15	.3
Type of admission						
Elective	–	–		–	–	
Nonelective	1.27	1.14, 1.42	<.001	1.35	1.12, 1.63	.001
Hospital bed size						
Small	–	–		–	–	
Large	1.15	1.08, 1.24	<.001	1.02	0.90, 1.16	.7
Medium	1.15	1.07, 1.24	<.001	1.05	0.87, 1.27	.6
Hospital teaching status						
Metropolitan, nonteaching	–	–		–	–	
Metropolitan, teaching	0.98	0.93, 1.03	.4	0.99	0.90, 1.10	.9
Nonmetropolitan	0.77	0.69, 0.85	<.001	0.67	0.54, 0.83	<.001
Insurance						
Medicaid	–	–		–	–	
Medicare	0.95	0.90, 1.00	.056	0.98	0.86, 1.11	.7
Other	0.68	0.63, 0.73	<.001	0.70	0.58, 0.85	<.001
Private	0.73	0.69, 0.77	<.001	0.83	0.71, 0.96	.014
Hypertension						
No	–	–		–	–	
Yes	1.01	0.96, 1.05	.7	1.04	0.96, 1.14	.3
Diabetes						
No	–	–		–	–	
Yes	1.11	1.05, 1.17	<.001	1.12	1.02, 1.23	.017
Coagulopathy						
No	–	–		–	–	
Yes	0.98	0.94, 1.02	.3	0.88	0.80, 0.97	.007
Anemia						
No	–	–		–	–	
Yes	1.00	0.95, 1.04	>.9	1.06	0.97, 1.15	.2
Chronic pulmonary disease						
No	–	–		–	–	
Yes	1.05	0.99, 1.11	.10	1.07	0.97, 1.19	.2
Congestive heart failure						
No	–	–		–	–	
Yes	0.98	0.91, 1.05	.6	0.93	0.84, 1.02	.10
Alcohol abuse						
No	–	–		–	–	
Yes	0.92	0.88, 0.96	<.001	1.01	0.93, 1.09	.9
Drug abuse						
No	–	–		–	–	
Yes	1.10	1.02, 1.18	.011	1.24	1.06, 1.45	.006
Depression						
No	–	–		–	–	
Yes	1.03	0.97, 1.10	.4	1.10	0.99, 1.23	.085
Obesity						
No	–	–		–	–	
Yes	0.91	0.86, 0.97	.005	0.91	0.82, 1.02	.10
Fluid and electrolyte disorders						
No	–	–		–	–	
Yes	1.14	1.09, 1.19	<.001	1.04	0.95, 1.13	.4
Ascites						
No	–	–		–	–	
Yes	1.56	1.48, 1.63	<.001	1.26	1.13, 1.41	<.001
Esophageal varices						
No	–	–		–	–	
Yes	0.82	0.78, 0.86	<.001	1.00	0.90, 1.10	>.9
Portal hypertension						
No	–	–		–	–	
Yes	0.95	0.91, 0.99	.008	0.99	0.91, 1.07	.8
Hepatorenal syndrome						
No	–	–		–	–	
Yes	0.82	0.74, 0.91	<.001	0.96	0.84, 1.09	.5
Sepsis						
No	–	–		–	–	
Yes	0.56	0.49, 0.64	<.001	0.51	0.39, 0.66	<.001
Discharged to nonhome setting						
No	–	–		–	–	
Yes	0.78	0.74, 0.82	<.001	0.71	0.65, 0.78	<.001
Length of stay	1.00	0.99, 1.00	.014	0.99	0.99, 1.00	.006

*P* for interaction (acute kidney injury × CKD): <.001.

CI = confidence interval, CKD = chronic kidney disease, OR = odds ratio.

## 4. Discussion

In this national analysis of 1,68,816 weighted index hospitalizations for cirrhosis in the 2016 to 2017 NRD, AKI complicated 25.6% of admissions, and the overall 30-day unplanned readmission rate was 25.0%. Readmissions were more frequent after admissions with AKI than without AKI (26% vs 24%), and AKI remained independently associated with higher odds of 30-day readmission after adjustment (OR 1.10, 95% CI: 1.05–1.16). The magnitude of this adjusted association was modest, corresponding to an absolute difference of 1.8 percentage points, and was smaller than the associations observed for ascites (OR 1.52) and chronic kidney disease (OR 1.42) in the same model. Given the size of the analytic sample, statistical significance alone conveys little about clinical importance, and the finding is best interpreted at the population rather than the individual level. Index admissions with AKI also reflected higher resource use and more complex discharge needs, including longer LOS, higher charges, and more frequent nonhome discharge.

Our results are consistent with prior national and multistate literature showing that early unplanned readmission after cirrhosis hospitalization is frequent and varies across data sources, coding eras, and cohort definitions.^[4-6,22]^ The association between index-admission AKI and early readmission is clinically credible. Large population analyses and contemporary hospitalized cohorts have shown that AKI in cirrhosis is associated with longer hospital stays, higher costs, and higher short-term mortality, consistent with AKI marking severe decompensation and care complexity rather than an isolated event.^[23-25]^

Several noncausal pathways may explain this association. AKI may mark patients with residual hemodynamic instability and systemic inflammation that is incompletely resolved at discharge, and it can narrow outpatient management options by limiting diuretic titration and lowering tolerance for modest shifts in volume status or infection burden. Consensus recommendations, therefore, emphasize standardized AKI recognition and evaluation.^[9]^ Contemporary mechanistic frameworks describe acute decompensation as a systemic process in which inflammation, circulatory dysfunction, and organ cross-talk interact, making renal dysfunction a visible signal of broader multisystem fragility.^[26,27]^

Beyond AKI, the strongest predictors in our model reflect decompensation burden and reduced physiologic reserve. Ascites was the most prominent correlate of early readmission, consistent with evidence that volume-related complications generate recurrent acute care needs and depend on reliable outpatient monitoring and rapid access to procedures.^[7,12,28]^ Chronic kidney disease (CKD) was also associated with higher odds of readmission, consistent with baseline renal impairment reducing physiologic reserve and complicating guideline-consistent management of ascites, infection, and medication safety.^[7,28,29]^ Risk modeling work in high-risk cirrhosis subgroups shows the same clustering of vulnerability, with ascites-related care needs and kidney dysfunction recurring as major correlates of early return to the hospital.^[12]^

Baseline CKD modified the association between AKI and readmission. Among patients without CKD, AKI was associated with higher odds of readmission, whereas among patients with CKD, no association was evident. One explanation is that preexisting CKD already confers substantial readmission risk, so an acute decline superimposed on established kidney disease adds little further information, whereas in patients with previously normal function, AKI marks a new and potentially reversible departure. Coding of acute kidney injury (AKI) may also be less specific when chronic impairment is present. To our knowledge, this interaction has not been reported in prior readmission analyses in cirrhosis, and it requires confirmation.

Admission and hospital factors in our model point to system-level vulnerability. Nonelective and weekend admissions were associated with higher odds of readmission, patterns that plausibly reflect higher presenting acuity and constrained access to timely procedures, consultative services, and discharge coordination.^[5,6]^ Lower readmission odds in nonmetropolitan hospitals may reflect differences in patient selection, transfer practices, competing risks, or local admission thresholds rather than superior outcomes.^[5,6]^ These observations are consistent with readmission risk reflecting both clinical phenotype and care context, and raise the possibility that interventions spanning inpatient and outpatient settings would be required; this was not evaluated here.

Insurance type remained associated with readmission risk in our model, with Medicaid coverage carrying higher odds than private or other insurance, consistent with evidence that socioeconomic disadvantage is linked to higher readmission risk in decompensated cirrhosis.^[10]^ Household income quartile was not consistently associated with readmission after adjustment, and the baseline income distribution differed only trivially by AKI status. Insurance-related gradients may reflect differences in access to timely outpatient follow-up, medication affordability, transportation, and caregiver support, which become especially consequential when AKI or advanced ascites calls for close surveillance.^[7,9,10,29]^ Early outpatient follow-up after cirrhosis hospitalization has been associated with improved short-term outcomes.^[30]^

These observations generate the testable hypothesis that readmission-reduction efforts could be directed toward high-risk clinical phenotypes, particularly cirrhosis complicated by AKI. Guideline-consistent components might include a clear diuretic and volume plan, early post-discharge monitoring of kidney function and electrolytes, medication reconciliation to limit nephrotoxin exposure, and structured surveillance for infection and encephalopathy recurrence.^[7,9,29]^ Structured transitional care programs have reported reductions in early readmission and cost in decompensated cirrhosis,^[31]^ and app-supported post-discharge monitoring has been evaluated after hepatic encephalopathy hospitalization.^[32]^ Neither was evaluated in an AKI-defined population, and the present data cannot establish that such approaches would be effective in this group.

Inverse associations observed for certain severe complications should be interpreted cautiously and not as protective effects; severe infection and advanced decompensation are strongly associated with short-term mortality, which reduces the opportunity for readmission among the sickest patients.^[7,25,33]^ Lower readmission odds among patients discharged to nonhome settings may similarly reflect structured monitoring rather than lower underlying risk.

The principal strengths of this study are its use of a large, all-payer national dataset in the ICD-10 era, survey-weighted estimation, a focus on unplanned rather than all-cause readmission, and the inclusion of time-to-readmission and readmission-level outcomes.

## 5. Limitations

Several limitations should be considered. First, exposure and case ascertainment rely on administrative coding. ICD-10 codes are insensitive for biochemically defined AKI, identifying only 26% to 36% of episodes in a validation cohort,^[34]^ so mild injury is under-ascertained, and the observed association is likely conservative. Requiring cirrhosis as the principal discharge diagnosis also limits generalizability. Second, the NRD does not distinguish AKI present on admission from AKI arising during the hospitalization, and these differ in precipitants, reversibility, and prognosis.

Third, the NRD lacks MELD and Child-Pugh scores, AKI stage (KDIGO 1–3) and trajectory, laboratory values, specialist input such as nephrology involvement, and treatment details. Because AKI was identified by ICD-10-CM code alone without staging, the exposure does not separate mild, self-limited AKI from severe or persistent AKI, which likely carry different readmission risks. Residual confounding by disease severity is therefore likely, and an OR of 1.10 is consistent with a substantial contribution from it. Without treatment data, we also cannot tell whether readmission reflects the AKI episode itself or gaps in post-discharge management.

Fourth, discharge disposition and index LOS are measured after AKI ascertainment and may lie on the causal pathway between AKI and readmission, so adjusting for them risks mediator adjustment rather than confounder adjustment. In a sensitivity analysis excluding both, the AKI estimate was slightly lower (adjusted OR 1.06 vs 1.10) and remained significant.

Fifth, patients who die during the index admission cannot be readmitted, and because AKI is associated with higher inpatient mortality^[23-25]^ this competing risk would bias the association toward the null. A competing-risk analysis was not feasible because out-of-hospital death is not captured. Finally, the NRD calendar-year structure prevents capture of readmissions across years, emergency department visits not resulting in admission are not recorded, and HCUP reporting rules restrict presentation of small cell counts.

## 6. Conclusion

Among adults hospitalized for cirrhosis, AKI during the index admission was independently associated with unplanned 30-day readmission, although the magnitude of this association was modest, and marked a subgroup with higher resource use and more complex discharge needs. The association was confined to patients without preexisting chronic kidney disease. These findings generate the hypothesis that risk-stratified transitional care prioritizing early post-discharge reassessment and kidney- and volume-focused monitoring could reduce early readmission in this group.^[7,9,29-32]^ Prospective evaluation is required before such an approach can be recommended.

## Acknowledgments

All authors would like to extend their sincere regards to the team of the Research Council of Pakistan (RCOP) for their guidance and mentorship.

## Author contributions

**Conceptualization:** Asad Ali Ahmed Cheema, Eliza Aisha, Muhammad Munhib Shehzad.

**Data curation:** Asad Ali Ahmed Cheema, Eliza Aisha, Ayesha Naveed, Ayesha Wasim, Syed Muhammad Ibrahim Shah Bokhari, Yaseen Ali Khan, Fatima Jawed, Ahsan Raza Raja, Sajeela Khan.

**Formal analysis:** Asad Ali Ahmed Cheema, Eliza Aisha.

**Investigation:** Ayesha Naveed, Ayesha Wasim, Yaseen Ali Khan, Fatima Jawed, Muhammad Usman Hashmi, Haider Imran.

**Methodology:** Asad Ali Ahmed Cheema, Eliza Aisha, Muhammad Munhib Shehzad.

**Project administration:** Asad Ali Ahmed Cheema, Muhammad Munhib Shehzad.

**Resources:** Ayesha Naveed, Ayesha Wasim.

**Supervision:** Muhammad Munhib Shehzad, Syed Muhammad Ibrahim Shah Bokhari.

**Validation:** Syed Muhammad Ibrahim Shah Bokhari.

**Visualization:** Asad Ali Ahmed Cheema, Eliza Aisha, Ayesha Naveed, Ahsan Raza Raja.

**Writing – original draft:** Asad Ali Ahmed Cheema, Eliza Aisha, Muhammad Munhib Shehzad.

**Writing – review & editing:** Asad Ali Ahmed Cheema, Eliza Aisha, Muhammad Munhib Shehzad, Ayesha Naveed, Syed Muhammad Ibrahim Shah Bokhari, Muhammad Usman Hashmi.
